# Which behaviour change techniques are most effective in improving healthcare utilisation in COPD self-management programmes? A protocol for a systematic review

**DOI:** 10.1136/bmjresp-2018-000369

**Published:** 2019-04-11

**Authors:** Katelyn Rene Smalley, Lisa Aufegger, Kelsey Flott, Gracie Holt, Erik K Mayer, Ara Darzi

**Affiliations:** Surgery and Cancer, Imperial College London, London, UK

**Keywords:** chronic disease, health knowledge, attitudes, practice, patient participation, personal autonomy, self-assessment, health literacy, health behaviour, health promotion, risk reduction behaviour, motivation, problem solving, decision making, self administration, disease management

## Abstract

**Introduction:**

Self-management interventions are often presented as a way to improve the quality of care for patients with chronic illness. However, self-management is quite broadly defined and it remains unclear which types of interventions are most successful. This review will use the Theoretical Domains Framework (TDF) as a lens through which to categorise self-management interventions regarding which programmes are most likely to be effective and under which circumstances.

The aim of this study is to (1) describe the types of self-management programmes that have been developed in chronic obstructive pulmonary disease (COPD) and identify the common elements between these to better classify self-management, and (2) evaluate the effect that self-management programmes have on the healthcare behaviour of patients with COPD by classifying those programmes by the behaviour change techniques used.

**Methods and analysis:**

A systematic search of the literature will be performed in MEDLINE, EMBASE, HMIC and PsycINFO. This review will be limited to randomised controlled trials and quasi-experimental studies. The review will follow PRISMA-P guidelines, and will provide a PRISMA checklist and flowchart. Risk of bias in individual studies will be assessed using the Cochrane Risk of Bias criteria, and the quality of included studies will be evaluated using the GRADE criteria, and will be reported in a Summary of Findings table.

The primary analysis will be a catalogue of the interventions based on the components of the TDF that were used in the intervention. A matrix comparing included behaviour change techniques to improvements in utilisation will summarise the primary outcomes.

**Ethics and dissemination:**

Not applicable, as this is a secondary review of the literature.

**Prospero registration number:**

CRD42018104753.

**Key messages**

- The ability of self-management programmes to change behaviour has implications for healthcare utilisation.
- Understanding the evidence on self-management programmes’ ability to change behaviour will enable improvements to such programmes in the future.
- This systematic review will connect behaviour change techniques to observed changes in behaviour in the context of a highly complex intervention—self-management programmes for chronic obstructive pulmonary disease.

**Strengths**

- This study will catalogue the types and number of self-management programmes available globally for the chronic condition selected for in-depth review.
- The review will analyse self-management programmes based on the association between the design of the intervention and its effect on outcomes, which will enable the development of future evidence-based programmes.
- This review will analyse programmes based on the Theoretical Domains Framework, which will enable comparison across types of interventions in a systematic way.
- By focusing on utilisation as an outcome measure, this review analyses the proposed mechanism of action of self-management programmes, namely behaviour change.

**Limitations**

- Potential limitations of this study include the heterogeneity of measures and outcomes evaluated and the potentially reduced number of studies in subgroup analyses, which may negatively influence the statistical power in data synthesis.

## Introduction

Self-management interventions are often presented as a way to improve the quality of care for patients with chronic illness.^1–3^ These programmes rely on patients assuming greater responsibility for both managing their own health outside of the healthcare setting and increasing their sophistication in interacting with healthcare providers. Patients are being recognised as an underused resource in the struggle to achieve high-value, appropriate care. Often referred to as the Triple Aim, this framework considers healthcare improvement along three domains: improving the experience of care, improving the health of populations and reducing per capita costs of healthcare.^4^ A variety of patient education programmes for chronic conditions, including simple informational resources, patient training and active management of conditions by patients, have been shown to improve outcomes, be highly desirable for patients and to reduce costs in certain circumstances. For instance, Pinnock *et al* found that across 24 randomised controlled trials (RCTs), the reduction in hospitalisations associated with self-management programmes for asthma offset the cost of implementation of those programmes.^5^ Furthermore, improving health literacy through education has been proven to increase engagement and improve health outcomes.^6^ What remains unclear is which types of interventions are most successful in achieving the Triple Aim by changing the way healthcare is consumed and which situations are most amenable to these types of interventions.

The body of literature trialling self-management programmes is extensive, and it is important to evaluate the success of these programmes such that they are as comparable as possible with one another. In order to achieve a robust comparison between programmes, included studies must contain small variations in specific attributes such as training components, target population and outcomes reported. This approach was chosen so that this analysis has the potential for transferability. This study included a first stage during which a long-term condition was selected for systematic examination. Based on the number of studies in this first phase, chronic obstructive pulmonary disease (COPD) was selected as the subject of this review (n=946 out of 11 665). With a prevalence of 1.2 million, COPD is the most common respiratory disease in the UK.^7^ Because COPD is (1) widely studied as a condition amenable to self-management and (2) quite prevalent, a review of self-management programmes for this disease will yield a robust dataset from which principles of self-management education can be gleaned.

COPD is more precisely characterised as a syndrome rather than a single condition, in that both symptoms and underlying pathology can vary quite widely. COPD is primarily composed of chronic bronchitis (narrowing of the airways) and emphysema (breakdown of air sacs), both of which make respiration more difficult.^7^ While generally associated with smoking, not all smokers develop COPD. Since airway obstruction also has multiple causes, experts suspect a genetic component to the disease.^8^ Treatment of COPD is primarily through inhalers for routine maintenance and antibiotics for treating exacerbations. Non-pharmaceutical interventions like pulmonary rehabilitation and smoking cessation programmes often complement the medical regimen.^9^

A 2014 systematic review of reviews evaluated the suitability of several long-term conditions for self-management programmes, and COPD was among those identified as most amenable to self-management.^10^ For chronic diseases, such as COPD, asthma and type 2 diabetes, self-management has the potential to improve symptoms or alter the course of the disease.^10^ Historically, self-management programmes in respiratory disease focused primarily on asthma; however, in recent years, these programmes have been adapted to COPD. Most commonly, these programmes comprise an action plan for symptom management and a ‘rescue pack’ of antibiotic medication in the event of an exacerbation. Educational resources and access to a nurse specialist via phone are also common features. As described in further detail below, this review will evaluate changes to healthcare utilisation for patients with COPD with respect to the presence or absence of those features.

### Context

Underpinning the popularity of self-management programmes is the assumption that this particular type of education will lead to changes in behaviour that will result in better health and thus reduced need for healthcare. The key feature of self-management programmes is to provide tools to assist patients in changing their behaviour and taking a larger role in their own healthcare, in contrast to programmes that provide mere education or skills training.^10^ Behaviour change, both in terms of lifestyle management and decisions on how to access healthcare, is especially essential for patients whose conditions require intensive management—that is to say, chronic illnesses with frequent exacerbations or proneness to infection.^11^ For the sake of clarity, this systematic review will focus on programmes that measure improvements in healthcare service use per se, and will set aside programmes that measure lifestyle changes only, such as those aiming to bolster physical activity.

Self-management is challenging to characterise because of the complexity of several of its components, and the lack of agreement around what self-management entails. Definitions vary widely in terms of both self-management and utilisation, and programmes can isolate one single long-term condition or introduce skills that are applicable across a range of conditions.^10–12^ Self-management can refer to a wide range of activities, including exercise, symptom monitoring and asking follow-up questions in healthcare appointments.^13^ The techniques used to encourage behaviour change can also vary widely; educational materials, action plans, email reminders and peer support are a (non-exhaustive) list of techniques that have been used.^10^

Other key concepts are also subject to complexity and variation. Utilisation can be measured in terms of cost, hospitalisations, prescriptions filled and office visits, to name a few. Self-management programmes can target patients with a particular chronic condition, multiple chronic conditions or can be available for a more generic population.^13^ The interaction of all of these complex elements makes it difficult to isolate best practices. Certain techniques, for instance educational modules and remote monitoring, may be important components of self-management programmes, but are not considered self-management per se.^10^ Further, concepts such as self-care (a broader concept including both self-management of disease and maintaining a healthy lifestyle), self-monitoring and self-treatment (both narrower constructs referring to managing specific aspects of illness at home) overlap significantly with self-management and in some cases have been used interchangeably.^3^ For the purposes of this review, a self-management programme is one which provides patients with knowledge, skills and expertise with respect to managing both their interactions with the healthcare system, and mitigating the effects of their disease while at home.

Assumptions about the level of patient expertise are inherent in the emphasis on self-management, health literacy and patient activation, but the nature and extent of that expertise is rarely made explicit. Sociodemographic factors like age, education and ethnicity are often used to explain variations in capacity to self-manage, but implicit in the popularity of these programmes is an understanding that there is a basic level of expertise that all patients must possess in order to comply with medical advice.^14^

The goal of many programmes is to surpass mere compliance and encourage patients to take a more active role in their care. However, Boehmer *et al* note that capacity to do so is dynamic and dependent on factors outside the individual.^15^ For instance, social capital and the ability to mobilise resources to support one’s care is critical, but so is a belief in one’s own self-efficacy.^16^ Thus, there is a recognition that education alone is not enough. Previous reviews have examined the associations between self-management performance and these psychosocial dimensions,^17^ but have not drawn the explicit link to the kind of behaviour changes that lead to changes in resource utilisation.

### Theories of behaviour change

Health services interventions, in contrast to other aspects of healthcare delivery like pharmaceutical trials, are highly complex.^18^ Thus, in recent years, greater focus has been placed on understanding the theory and mechanism of action behind a proposed intervention in order to maximise its chances of success. As Eccles *et al* report in their discussion of the use of theory to promote the uptake of research findings, “there may be important differences in the context and barriers between studies that assessed supposedly homogeneous interventions (107).”^19^ They point out that this constitutes a potentially expensive and fruitless form of trial and error in the quest for effective interventions, which perhaps underestimates the risks to efficiency, mortality and quality of life.

For this reason, this review considers a relatively homogeneous group of studies and attempts to disentangle the mechanisms of action, other behavioural changes and overarching contextual factors that contribute to variation in results. As Eccles *et al* describe, some mediators of behaviour (e.g. age, intelligence, geography) are largely immutable.^19^ However, health systems, especially the UK’s National Health Service, express a commitment to health equity in addition to quality and safety. At the root of health services research is a focus on improvement.^20 21^ Thus, in this context, as compared with other social sciences, it is important to consider theories that undertake to explain *change* in behaviour, rather than merely describe the current state of affairs.

A number of frameworks have been developed to characterise the principal mechanisms by which to change an individual’s behaviour, especially with respect to healthcare.^6 22–25^ Most prominent in the current literature is the Theoretical Domains Framework (TDF).^26^ The TDF is a synthesis of 33 theories of behaviour change and is rooted in past work on a Behaviour Change Wheel (BCW) that connects psychological and environmental factors to interventions in order to catalogue behaviour change techniques. The BCW is founded on a ‘behaviour system’ such that *capability*, *opportunity* and *motivation* interact to produce *behaviour* (COM-B). The COM-B system connects to interventions and policy levers around the BCW that give a theoretical basis for behaviour change interventions.^27^ The TDF expands on this framework to incorporate findings from other theoretical and empirical research, to yield 14 domains of behaviour change, which are composed of 93 behaviour change techniques (BCTs).^26 28^ This review will use the TDF as a lens through which to categorise the self-management interventions in the included studies. Assigning interventions by type will allow conclusions to be drawn regarding the self-management programmes most likely to be successful and under which circumstances.

In a recent systematic review of reviews, Newham *et al* found that self-management interventions for people with COPD generally improved health-related quality of life and reduced emergency visits.^29^ By coding interventions in the included studies by BCTs, they also discovered that BCTs addressing mental health led to greater improvements in those outcomes.^29^ This review will build on that previous work in a number of ways. First, the forthcoming review will add granularity to the findings of Newham *et al* by returning to the results of the primary studies, and using a more current and comprehensive search strategy. Second, this report will build on the BCT categorisation by locating the techniques within the TDF. This will contribute further to the understanding of the BCTs used, by evaluating their relationships to one another and other behavioural constructs. Finally, the meta-analysis of a variety of utilisation metrics, including ED visits, will provide insight into objective changes in behaviour that can be attributed to self-management interventions.

## Research aims

The aim of this study is to explain the variation in effectiveness of self-management programmes, by classifying those programmes by the behaviour change techniques used, and subsequent effects on patients’ healthcare utilisation patterns. Where possible, meta-analysis will be used to quantify the effects of these programmes on utilisation, for instance, changes in hospital admissions and emergency visits.

## Methods

### Search strategy

A systematic search of the literature will be performed in MEDLINE, EMBASE, HMIC and PsycINFO. Search strings (table A, online supplementary file 1) will combine free terms and controlled vocabulary, whenever supported. In preparing the search strings, previous systematic reviews on the topic were consulted to ensure a breadth of relevant potential studies for inclusion.

10.1136/bmjresp-2018-000369.supp1 Supplementary data

Language restrictions will be applied and articles in English will be included. The search will be limited to the 20-year period from 1998 to 2018 to ensure the relevance of the studies. The reference lists of relevant articles will also be screened to ensure all eligible studies are captured. Authors of protocols potentially meeting inclusion criteria as registered in PROSPERO will be contacted to provide further information about the progress of the corresponding study.

Preliminary research suggests that the body of literature on self-management programmes is substantial, and there is wide variation in the nature of these programmes. For these reasons, based on the scoping stage of the review, COPD was selected to review systematically.

### Study selection criteria

A summary of the participants, interventions, comparators, outcomes and study design is provided in table 1. Generally speaking, the study will consider self-management and expert patient interventions for adult patients with chronic illnesses that require frequent interaction with the healthcare system. This review will be limited to RCTs and observational studies with a quasi-experimental design and control group. Due to the nature of the outcome of interest (utilisation rates), only studies that provide quantitative data will be included.

**Table 1:** Inclusion and exclusion criteria

	Included	Excluded
**Participants**	Phase 1—scoping review: adult patients with one or more chronic condition (as defined in Taylor *et al*,^10^ reproduced in online supplementary material, table E) Phase 2—systematic review: adult patients with COPD	Patients who do not reside independently in the community Patients younger than 18 years Patients with dental conditions Interventions directed at parents/caregivers Interventions targeted at clinicians Interventions targeted at organisations
**Intervention**	Self-management programme Patient activation programme Expert patient programme Health literacy programme Disease management Integrated management	Self-taught self-management Patient education without self-management component Case management Cognitive-behavioural therapy Medication therapy management Electronic reminders Pharmaceutical clinical trial Specific to COPD: Physical activity Exercise Pulmonary rehabilitation Remote monitoring
**Comparator**	Usual care (no intervention) Head-to-head comparison of two interventions in same population	No control group
**Outcomes**	Primary: Change in healthcare utilisation Total cost of care Hospitalisations ED visits Readmissions GP/outpatient visits Exacerbations Other Secondary: Change in quality of life Change in PROMs/PREMs Mortality	Intention to change behaviour Self-efficacy Patient activation Health literacy Medication adherence Compliance Physical activity Self-reported outcomes
**Study design**	Randomised controlled trial Interrupted time series Difference in differences Other quasi-experimental designs	Case–control Case study Ethnography Qualitative results only Systematic/scoping review Protocol—no results Feasibility study

COPD, chronic obstructive pulmonary disease; ED, emergency department; GP, general practitioner; PREM, patient-reported experience measure; PROM, patient-reported outcome measure.

The first phase of the review is agnostic to whether the programmes include disease-specific education, general self-management skills or address patients with multiple chronic conditions. However, because of the breadth of literature available on the topic of self-management, a first-stage scoping review was conducted in order to select an appropriate long-term condition to review in depth; COPD was selected because a plurality of studies that otherwise met exclusion criteria focused on this condition.

Under Cochrane guidelines, exclusion of studies on the basis of availability of outcome data is not preferred except in certain circumstances. However, the primary objective of this review is to attempt to understand the effect that different self-management programme designs have on utilisation. For this reason, it is important to exclude primary studies that do not measure utilisation as an outcome. Nonetheless, the authors recognise that other outcomes are of substantial interest to both patients and policy-makers, and therefore measures of health-related quality of life, disease management, mortality, and patient-reported outcomes and experience will be included as secondary outcomes.

For studies that do not fit easily across the inclusion/exclusion criteria, if subgroup results are provided, then the group that meets the inclusion criteria will be included. Otherwise, the study will be excluded in full. Further, studies that provide insufficient detail about the intervention (eg, components of the intervention and/or self-management have not been defined) will be excluded. Since a behaviour change technique analysis is key to this review, studies that do not describe programme details cannot be included. In cases in which programme components are specified but can be interpreted in multiple ways, the authors of the original papers will be contacted for clarity.

### Screening and data extraction

Data extraction will take place in duplicate, using a modified version of the Cochrane EPOC’s Good Practice Data Collection form for Intervention Review (randomised and non-randomised trials).^30^ Data will be managed in Mendeley, and data extraction and decisions will be recorded in Excel.

Initial screening of studies will be based on the information contained in their titles and abstracts and will be conducted by two independent investigators (KRS and GH). Full-paper screening will be conducted by KRS, and a 10% sample will be reviewed by LA. Inclusion and exclusion decisions will be made by following the full text selection process (table B, online supplementary material). Cohen’s kappa will be used to measure intercoder agreement in each screening phase. When there are doubts regarding inclusion or exclusion, a third investigator will be involved in the decision (EKM).

The same two independent investigators (KRS and LA) will extract information from the included studies into a standardised form. The data collected for each study will include name of the first author, year of publication, technology, intervention components and characteristics, study duration, participants’ and setting characteristics, outcomes and retention rates. Two investigators will review the abstraction form for consistency (KRS and LA). Disagreements will be resolved by a third investigator (EKM).

### Quality assessment

Risk of bias in individual studies will be assessed using the Cochrane Risk of Bias criteria, as reproduced in table C (online supplementary material). Each study will be evaluated as having a high, neutral or low risk of bias via selection, performance, detection, reporting and/or attrition.

The quality of included studies will be evaluated using the GRADE criteria and will be reported in a Summary of Findings table as modelled in table D (online supplementary material). Following the Cochrane convention, RCTs will be assumed to have a ‘high’ quality of evidence, which may be downgraded based on various facets of study design, and observational studies will be presumed to have a ‘low’ quality of evidence that can either be downgraded to ‘very low’ or upgraded to ‘moderate’ or ‘high’ based on characteristics of the study.

Two independent reviewers will score the selected studies based on these criteria (KRS and LA) and provide justifications. Disagreements will be resolved through discussion with a third person (EKM).

### Analysis

Of principal interest in this review are the behaviour change techniques that underpin various self-management education programmes, and the ability of these techniques to change patterns of use of the healthcare system by chronically ill patients. Thus, the primary analysis will be a catalogue of the interventions based on the components of the TDF that were used in the intervention.^26^ For instance, study X provided *feedback on behaviour* and *information about health* consequences, but not *social support*. The aim of this analysis is to characterise self-management programmes based on the techniques that seem most effective in motivating changes in utilisation. The analysis will be completed as a three-step process. To account for variation in the way that outcome measures have been defined, first the outcomes will be simplified such that each intervention will be categorised as having an observable reduction in utilisation, an increase in utilisation or no statistically significant change. Then, the BCTs used in each study will be extracted and summarised in a separate table. Finally, associations will be drawn between the observed elements of the TDF and the observed change in behaviour.

Self-management is a complex intervention, for which both programme design and outcomes can be widely defined. Where possible, these components will be used to complete a meta-analysis of the effects on utilisation outcomes. The aim of this analysis is to characterise self-management programmes based on the techniques that seem most effective in motivating changes in utilisation. The meta-analytic portion of the analysis will strive to consider a subset of outcomes that are as comparable as possible, especially in terms of the patient population under study and the metric of utilisation. As described in the Summary of Findings template (table D, online supplementary material), the analysis will accommodate for different measures of utilisation as different outcomes. That is to say, studies that measure utilisation by rate of hospitalisation will not be pooled with utilisation expressed as costs in the meta-regression. Studies that cannot be included in the meta-analysis will not be discarded, but instead will be described in the narrative synthesis only.

The narrative synthesis of the studies will describe the range of self-management interventions in terms of programme design, target population and setting, behaviour change techniques used and the generalisability of these findings. Secondary outcomes, including quality of life and patient-reported outcomes, will be described qualitatively.

### Subgroup and sensitivity analyses

Subgroup analyses will be conducted based on various patient characteristics, including age, socioeconomic status, education and/or race/ethnicity where possible. A matrix comparing included behaviour change techniques to changes in utilisation will be created.

Robustness checks will be performed by altering the control variables in the specifications of the meta-regression.

### Amendments

Any amendments to this protocol will be documented with reference to saved searches and analysis methods, which will be recorded in bibliographic databases (Ovid), Mendeley, and Excel templates for data collection and synthesis.

## Discussion

A prominent component of the move to higher-value healthcare (better quality at lower cost) has been to encourage patients to assume a larger role in providing and managing their care, whether by improving their health literacy, participating in shared decision-making with their doctors or engaging in self-management.^31^ Self-management has been a particularly attractive tool as it is recognised that many of the contributing factors to health occur outside the healthcare setting.^10^ Self-management education then is a tool to both promote healthy lifestyle behaviours that reduce the need for care in the first place, but also (especially for patients with long-term illness) to teach how to access healthcare services most appropriately, and to delegate certain care activities to patients themselves.

While previous systematic reviews have evaluated these programmes for their effects on patient experience, quality of life and clinical outcomes,^10 32–34^ the contribution of this review will be the emphasis on programmes’ ability to change behaviour in the ways theories have proposed. Thus, the objective of this review is to evaluate the behaviour change techniques used by various self-management programmes and their observed effects on utilisation of the healthcare system.

COPD is a condition for which clinical, social and environmental factors are closely linked, in contrast to other respiratory conditions such as bronchiectasis or cystic fibrosis. For example, COPD is highly associated with lifestyle choices such as smoking, and environmental challenges such as high levels of pollution, which are concentrated in patients of lower socioeconomic status.^6 8^ Presumably, this context will influence the ways that COPD self-management programmes specifically have been developed and implemented. Nonetheless, overlap exists in the self-management techniques that may be useful across chronic respiratory diseases. While COPD has been the most widely studied respiratory disease in terms of self-management, categorising the techniques used by self-management programmes according to the TDF will allow for common themes and general principles of self-management to emerge. Thus, the lessons learnt from this review will be more widely applicable to diseases (especially other respiratory diseases) that have been less well represented in the literature.

## Reporting findings

We will use the PRISMA-P checklist when writing this report.^35^
