# Supplementary material for: Which behaviour change techniques are most effective in improving healthcare utilisation in COPD self-management programmes? A protocol for a systematic review
Source: BMJ Open Respir Res. 2019 Apr 11;6(1):e000369. doi: 10.1136/bmjresp-2018-000369 (PMC6530545; doi:10.1136/bmjresp-2018-000369)
Supplement: Supplementary data [file bmjresp-2018-000369supp001.pdf]

Online only supplementary material
Which behaviour change techniques are most effective in improving healthcare utilisation in COPD self-management programmes?

Table A. Concepts and search items	
**Database**	**Search items**
**MEDLINE** (via Ovid)	**Self-Management and Expert Patient Programmes**<br>1. patient education as topic/ or health education/ or health knowledge, attitudes, practice/<br>2. patient participation/ or consumer participation/ or Personal Autonomy/ or self-assessment/<br>3. Health Literacy/<br>4. health behavior/ or health promotion/ or risk reduction behavior/ or (risk adj3 reduc* adj3 behav*).tw. or (health adj5 (promot* or educat* or behav*)).tw.<br>5. motivation/ or goals/ or problem solving/ or exp decision making/<br>6. self administration/<br>7. patient compliance/ or disease management/<br>8. ((patient* or consumer* or client*) adj5 (educat* or participat* or behaviour* or behavior* or compliance or cent*red or activat*)).tw.<br>9. ((patient* or consumer* or client*) adj5 manag* adj5 disease*).tw.<br>10. (((behav* adj3 chang*) or (problem* adj3 solv*) or (goal* adj3 setting) or (decision* adj3 mak*)) adj5 (patient* or consumer* or client*)).tw.<br>11. (self adj management).ti,ab.<br>12. ((patient*1 or consumer*1 or client*1) adj4 (train*3 or teach*3 or instruct*3 or skill*3)).ti,ab.<br>13. (expert adj patient).ti,ab.<br>14. (personal* budget).ti,ab.<br>15. (non adj (professional*1 or medic*2)).ti,ab.<br>16. 1 or 2 or 3 or 4 or 5 or 6 or 7 or 8 or 9 or 10 or 11 or 12 or 13 or 14 or 15<br><br>**Chronic Conditions**<br>17. exp Chronic Disease/<br>18. chronic.ti,ab.<br>19. (chronic adj (illness*2 or disease* or condition*)).ti,ab.<br>20. (long adj term adj (illness*2 or disease*1 or condition*1)).ti,ab.<br>21. 17 or 18 or 19 or 20<br>22. 16 and 21 [Self-management programmes AND Chronic Conditions]<br><br>**Study Design**<br>23. ("quasi-experiment$" or quasiexperiment$ or "quasi random$" or quasirandom$ or "quasi control$" or quasicontrol$ or ((quasi$ or experimental) adj3 (method$ or study or trial or design$))).ti,ab,hw.<br>24. ("time series" adj2 interrupt$).ti,ab,hw.<br>25. trial.ti. or ((study adj3 aim?) or "our study").ab.<br>26. (clinical trial or multicenter study).pt.<br>27. (multicentre or multicenter or multi-centre or multi-center).ti.<br>28. random$.ti,ab. or controlled.ti.<br>29. (control adj3 (area or cohort? or compar? or condition or group? or intervention? or participant? or study)).ab. not (controlled clinical trial or randomized controlled trial).pt.<br>30. 23 or 24 or 25 or 26 or 27 or 28 or 29

	**Exclusions**<br>31. "comment on".cm. or systematic review.ti. or literature review.ti. or editorial.pt. or letter.pt. or meta-analysis.pt. or news.pt. or review.pt. [to exclude irrelevant publication types]<br>32. exp animals/ not humans.sh.<br>33. 31 or 32<br><br>**Combined**<br>34. 22 and 30 [Self-management programmes AND Chronic Conditions AND Study Design]<br>35. 34 not 33 [Self-management programmes AND Chronic Conditions AND Study Design NOT Exclusions]<br>36. limit 35 to yr="1998 - 2018" [Self-management programmes AND Chronic Conditions AND Study Design NOT Exclusions; 1998-2018]
**EMBASE** (via Ovid)	**Self-Management and Expert Patient Programmes**<br>1. drug self administration/<br>2. patient compliance/ or patient education/ or patient participation/ or Personal Autonomy/<br>3. Health Literacy/<br>4. attitude to health/ or health behavior/ or health education/ or health promotion/<br>5. disease management/ or risk reduction/ or patient care plan/<br>6. motivation/ or problem solving/ or exp decision making/ or patient decision making/<br>7. ((patient* or consumer* or client*) adj5 (educat* or participat* or behaviour* or behavior* or compliance or cent*red or activat*)).tw.<br>8. (health adj5 (promot* or educat* or behav*)).tw.<br>9. (risk adj3 reduc* adj3 behav*).tw.<br>10. ((patient* or consumer* or client*) adj5 manag* adj5 disease*).tw.<br>11. (((behav* adj3 chang*) or (problem* adj3 solv*) or (goal* adj3 setting) or (decision* adj3 mak*) or coping) adj5 (patient* or consumer* or client*)).tw.<br>12. (patient adj education).ti,ab.<br>13. (self adj management).ti,ab.<br>14. ((patient*1 or consumer*1 or client*1) adj4 (train*3 or teach*3 or instruct*3 or skill*3)).ti,ab.<br>15. (expert adj patient).ti,ab.<br>16. (non adj (professional*1 or medic*2)).ti,ab.<br>17. (personal* budget).ti,ab.<br>18. 1 or 2 or 3 or 4 or 5 or 6 or 7 or 8 or 9 or 10 or 11 or 12 or 13 or 14 or 15 or 16 or 17<br><br>**Chronic Conditions**<br>19. exp chronic disease/<br>20. chronic.ti,ab.<br>21. (chronic adj (illness*2 or disease* or condition*)).ti,ab.<br>22. (long adj term adj (illness*2 or disease*1 or condition*1)).ti,ab.<br>23. 19 or 20 or 21 or 22<br>24. 18 and 23 [Self-management programmes AND Chronic Conditions]

	**Study Design**<br>25. *experimental design/ or *pilot study/ or quasi experimental study/<br>26. ("quasi-experiment$" or quasiexperiment$ or "quasi random$" or quasirandom$ or "quasi control$" or quasicontrol$ or ((quasi$ or experimental) adj3 (method$ or study or trial or design$))).ti,ab.<br>27. ("time series" adj2 interrupt$).ti,ab.<br>28. 25 or 26 or 27 or 28<br><br>**Exclusions**<br>29. (animal model? or animal experiment? or animal study? or animal trial? or canine or feline or bovine or cow or cows or mice or dog? or cat or cats or rabbit? or rat or rats or veterinar$).ti. or (animal or veterinary).hw.<br>30. (editorial or letter or note or "review" or trade or survey).pt.<br>31. 29 or 30<br><br>**Combined**<br>32. 24 and 28 [Self-management programmes AND Chronic Conditions AND Study Design]<br>33. 32 not 31 [Self-management programmes AND Chronic Conditions AND Study Design NOT Exclusions]<br>34. limit 33 to yr="1998 - 2018" [Self-management programmes AND Chronic Conditions AND Study Design NOT Exclusions 1998-2018]
**HMIC** (via Ovid)	**Self-Management and Expert Patient Programmes**<br>1. exp self management/ or exp self medication/<br>2. patient compliance/ or exp patient information/ or patient education/ or expert patients/<br>3. Health Literacy/<br>4. health behaviour/ or health education/ or patient knowledge/ or health promotion/<br>5. disease management/ or risk management/<br>6. exp motivation/ or exp Aims & objectives/ or problem solving/ or decision making/ or patient communication/<br>7. ((patient* or consumer* or client*) adj5 (educat* or participat* or behaviour* or behavior* or compliance or cent*red or activat*)).tw.<br>8. (health adj5 (promot* or educat* or behav*)).tw.<br>9. (risk adj3 reduc* adj3 behav*).tw.<br>10. ((patient* or consumer* or client*) adj5 manag* adj5 disease*).tw.<br>11. (((behav* adj3 chang*) or (problem* adj3 solv*) or (goal* adj3 setting) or (decision* adj3 mak*) or coping) adj5 (patient* or consumer* or client*)).tw.<br>12. (patient adj education).ti,ab.<br>13. (self adj management).ti,ab.<br>14. ((patient*1 or consumer*1 or client*1) adj4 (train*3 or teach*3 or instruct*3 or skill*3)).ti,ab.<br>15. (expert adj patient).ti,ab. or (personal* budget).ti,ab.<br>16. (non adj (professional*1 or medic*2)).ti,ab.<br>17. patient autonomy/ or Patient Participation/<br>18. expert patients/ or patient attitudes/ or patient choice/ or patient consent to treatment/ or patient management/ or patient reaction to treatment/<br>19. "self care of patient"/ or patient preparation/<br>20. 1 or 2 or 3 or 4 or 5 or 6 or 7 or 8 or 9 or 10 or 11 or 12 or 13 or 14 or 15 or 16 or 17 or 18 or 19

	**Chronic Conditions**<br>21. Chronic Disease/<br>22. chronic.ti,ab.<br>23. (chronic adj (illness*2 or disease* or condition*)).ti,ab.<br>24. (long adj term adj (illness*2 or disease*1 or condition*1)).ti,ab.<br>25. 21 or 22 or 23 or 24<br>26. 20 and 25 [Self-management programmes AND Chronic Conditions]<br><br>**Study Design**<br>27. ("quasi-experiment$" or quasiexperiment$ or "quasi random$" or quasirandom$ or "quasi control$" or quasicontrol$ or ((quasi$ or experimental) adj3 (method$ or study or trial or design$))).ti,ab,hw.<br>28. ("time series" adj2 interrupt$).ti,ab,hw.<br>29. trial.ti. or ((study adj3 aim?) or "our study").ab.<br>30. (clinical trial or multicenter study).pt.<br>31. (multicentre or multicenter or multi-centre or multi-center).ti.<br>32. random$.ti,ab. or controlled.ti.<br>33. (control adj3 (area or cohort? or compar? or condition or group? or intervention? or participant? or study)).ab. not (controlled clinical trial or randomized controlled trial).pt.<br>34. 27 or 28 or 29 or 30 or 31 or 32 or 33<br><br>**Exclusions**<br>35. "comment on".ti. or systematic review.ti. or literature review.ti. or editorial.pt. or letter.pt. or meta-analysis.pt. or news.pt. or review.pt. [to exclude irrelevant publication types]<br>36. exp animals/ not humans.sh.<br>37. 35 or 36<br><br>**Combined**<br>38. 26 and 34 [Self-management programmes AND Chronic Conditions AND Study Design]<br>39. 38 not 37 [Self-management programmes AND Chronic Conditions AND Study Design NOT Exclusions]<br>40. limit 39 to yr="1998 - 2018" [Self-management programmes AND Chronic Conditions AND Study Design NOT Exclusions 1998-2018]
**PsycINFO** (via Ovid)	**Self-Management and Expert Patient Programmes**<br>1. self administration/ or self-assessment/<br>2. patient compliance/ or patient education as topic/ or patient participation/ or Personal Autonomy/<br>3. Health Literacy/<br>4. attitude to health/ or health behavior/ or health education/ or health knowledge, attitudes, practice/ or health promotion/<br>5. disease management/ or risk reduction behavior/ or (risk adj3 reduc* adj3 behav*).tw.<br>6. motivation/ or goals/ or problem solving/ or exp decision making/<br>7. ((patient* or consumer* or client*) adj5 (educat* or participat* or behaviour* or behavior* or compliance or cent*red or activat*)).tw.<br>8. (health adj5 (promot* or educat* or behav*)).tw.<br>9. ((patient* or consumer* or client*) adj5 manag* adj5 disease*).tw.<br>10. (((behav* adj3 chang*) or (problem* adj3 solv*) or (goal* adj3 setting) or (decision* adj3 mak*) or coping) adj5 (patient* or consumer* or client*)).tw.

11. (patient adj education).ti,ab.
12. (self adj management).ti,ab.
13. ((patient*1 or consumer*1 or client*1) adj4 (train*3 or teach*3 or instruct*3 or skill*3)).ti,ab.
14. (expert adj patient).ti,ab. or (personal* budget).ti,ab.
15. (non adj (professional*1 or medic*2)).ti,ab.
16. 1 or 2 or 3 or 4 or 5 or 6 or 7 or 8 or 9 or 10 or 11 or 12 or 13 or 14 or 15

Chronic Conditions

17. exp "Chronicity (Disorders)"/ or exp Chronic Illness/
18. chronic.ti,ab.
19. (chronic adj (illness*2 or disease* or condition*)).ti,ab.
20. (long adj term adj (illness*2 or disease*1 or condition*1)).ti,ab.
21. 17 or 18 or 19 or 20
22. 16 and 21 [Self-management programmes AND Chronic Conditions]

Study Design

23. ("quasi-experiment$" or quasiexperiment$ or "quasi random$" or quasirandom$ or "quasi control$" or quasicontrol$ or ((quasi$ or experimental) adj3 (method$ or study or trial or design$))).ti,ab,hw.
24. ("time series" adj2 interrupt$).ti,ab,hw.
25. trial.ti. or ((study adj3 aim?) or "our study").ab.
26. (clinical trial or multicenter study).pt.
27. (multicentre or multicenter or multi-centre or multi-center).ti.
28. random$.ti,ab. or controlled.ti.
29. (control adj3 (area or cohort? or compar? or condition or group? or intervention? or participant? or study)).ab. not (controlled clinical trial or randomized controlled trial).pt.
30. 23 or 24 or 25 or 26 or 27 or 28 or 29

Exclusions

31. "comment on".cm. or systematic review.ti. or literature review.ti. or editorial.pt. or letter.pt. or meta-analysis.pt. or news.pt. or review.pt.
32. exp animals/ not humans.sh.
33. 31 or 32

Combined

34. 22 and 30 [Self-management programmes AND Chronic Conditions AND Study Design]
35. 34 not 33 [Self-management programmes AND Chronic Conditions AND Study Design NOT Exclusions]
36. limit 35 to yr="1998 - 2018" [Self-management programmes AND Chronic Conditions AND Study Design NOT Exclusions; 1998-2018]

Online only supplementary material
Which behaviour change techniques are most effective in improving healthcare utilisation in COPD self-management programmes?

**Table B. Full Text Selection Process**
1. Is the article published in English?<br>a. If yes, proceed to 2.<br>b. If no, exclude with code LANGUAGE.
2. Does the article describe an original, empirical study?<br>a. If yes, proceed to 3.<br>b. If commentary, editorial, or theoretical work, exclude with code STUDY DESIGN.
3. Is the article's study design a case-control, case study, ethnography, or other design that only yields qualitative results?<br>a. If yes, exclude with code STUDY DESIGN.<br>b. If no, proceed to 4.
4. Is the article's study design a high-quality quantitative design, such as RCT, ITS, DID, or other quasi-experimental approaches?<br>a. If yes, proceed to 5.<br>b. If no, exclude with code STUDY DESIGN.
5. Does the article articulate a clear control group/comparator?<br>a. If yes, proceed to 6.<br>b. If no, exclude with code COMPARATOR.
6. Does the article pertain to patients with chronic obstructive pulmonary disease, or report results for this population if a subset of a larger study?<br>a. If yes, proceed to 7.<br>b. If no, exclude with code PARTICIPANT.
7. Does the article pertain to patients who reside in an institutional setting?<br>a. If yes, exclude with code PARTICIPANT.<br>b. If no, proceed to 8.
8. Does the article pertain to patients with a primary diagnosis of mental illness, learning disability, or physical disability?<br>a. If yes, exclude with code PARTICIPANT.<br>b. If no, proceed to 9.
9. Does the article evaluate a **clearly-defined** self-management programme, health literacy programme, patient activation programme, expert patient programme, or disease-specific care management?<br>a. If yes, proceed to 10.<br>b. If no, exclude with code INTERVENTION.
10. Does the article evaluate self-taught self-management, patient education without self-management component, case management, a pharmaceutical trial, remote monitoring with a passive role for the patient, pulmonary rehabilitation, or physical exercise?<br>a. If yes, exclude with code INTERVENTION.<br>b. If no, proceed to 11.
11. Does the study include as an outcome a quantitative measure of healthcare utilisation?<br>a. If yes, INCLUDE and complete data extraction form.<br>b. If no, exclude with code OUTCOME.

Online only supplementary material
Which behaviour change techniques are most effective in improving healthcare utilisation in COPD self-management programmes?

**Table C. Risk of Bias**		
**Bias**	**Authors' Judgment**	**Support for Judgment**
Random sequence generation (selection bias)	[High, Low, or Unclear]	
Allocation concealment (selection bias)	[High, Low, or Unclear]	
Blinding of participants and personnel (performance bias)	[High, Low, or Unclear]	
Blinding of outcome assessment (detection bias)<br>- investigator-assessed	[High, Low, or Unclear]	
Blinding of outcome assessment (detection bias)<br>- self-reported	[High, Low, or Unclear]	
Incomplete outcome data (attrition bias)<br>- investigator-assessed	[High, Low, or Unclear]	
Incomplete outcome data (attrition bias)<br>- self-reported	[High, Low, or Unclear]	
Selective reporting (reporting bias)	[High, Low, or Unclear]	
Other bias	[High, Low, or Unclear]	

**Table D. Summary of Findings**						
**Comparison: Self-management programme v. usual care**						
**Patient or population:** people with long-term conditions that require intensive management<br>**Setting:** Outpatient, community, or clinic<br>**Intervention:** Self-management programme<br>**Comparison:** Usual care						
**Outcomes**	**Number of Participant Studies**	**Studies showing decrease**	**Studies showing increase**	**Studies with no significant result**	**Quality of Evidence (GRADE)**	**Comments**
Total Cost of care						
Hospitalisation						
A&E visits						
GP visits						
Length of stay						
OP visits						
ABX use						

GRADE: Grading of Recommendations Assessment, Development and Evaluation; A&E: accident and emergency; GP: general practitioner; OP: outpatient; ABX: antibiotic

Online only supplementary material
Which behaviour change techniques are most effective in improving healthcare utilisation in COPD self-management programmes?

**Table E. List of Chronic Conditions;** ***Taylor, et al. (2014), 'A rapid synthesis of the evidence on interventions supporting self-management for people with long-term conditions: PRISMS – Practical systematic Review of Self-Management Support for long-term conditions.'***			
1	Allergy/anaphylaxis	1	Addictions – substance and alcohol, etc.
2	Asthma	2	ADHD
3	Atrial fibrillation	3	Allergic rhinitis/rhinitis/sinusitis/rhinosinusitis
4	Childhood constipation	4	Amnesia
5	Chronic fatigue syndrome/ME	5	Amputations
6	Chronic kidney disease	6	Anaemia
7	Chronic obstructive pulmonary disease	7	Angina
8	Chronic pain	8	Angiooedema
9	Congestive heart failure	9	Anklosing spondylitis (and other arthritic conditions)
10	Dementia	10	Antenatal screening for haemoglobinoapthies – sickle cell and thalassemia, Downs
11	Depression	11	Anxiety and stress disorders (including complex and post-traumatic stress disorders)
12	Diabetes: Type I	12	Aphasia
13	Diabetes: Type II	13	Ataxia’s
14	Endometriosis	14	Autism
15	Epilepsy	15	Autoimmune disorders (e.g. lupus, Sjögrens syndrome)
16	Hypertension	16	Blood disorders
17	Hepatitis B	17	Brain injuries (including stroke and TIAs)
18	Hepatitis C	18	Bronchopulmonary dysplasia (chronic lung disease of infancy)
19	HIV	19	Burn injuries
20	Inflammatory bowel disease	20	Cancer
21	Irritable bowel disease	21	Cardiac arrhythmias
22	Low back pain	22	Cerebral palsy
23	Migraine	23	Crohn’s disease
24	Multiple sclerosis	24	Coeliac disease
25	Osteoarthritis	25	Connective tissue diseases
26	Parkinson’s disease	26	Coronary heart disease
		27	Cystic Fibrosis
		28	Digestive conditions, stomach ulcers, oesophagus, reflux
		29	Dizziness
		30	Dyslexia or dyspaxia
		31	Eating disorders (anorexia/bulimia)
		32	Eczema
		33	Endocrine disorders (thyrotoxicosis, hypothyroidism, hypogonadism, Cushing syndrome, Addison’s disease)
		34	Fibromyalgia/chronic widespread pain
		35	Gout
		36	Gynaecological problems, chronic pelvic pain
		37	Haemophilia and other coagulation disorders

		38	Heart failure
		39	Learning disabilities
		40	Lung fibrosis
		41	Lupus
		42	Malaria
		43	Medically unexplained symptoms
		44	Mood disorders (not only depression, but mania and bipolar disorders)
		45	Motor neurone disease
		46	Multimorbidity
		47	Multisystem autoimmune diseases (MSAIDs, including lupus)
		48	Muscular dystrophy(ies)
		49	Neuralgias (including, head and back pain)
		50	Newborn screening programme diseases, including thyroid disease, hearing loss
		51	Obesity
		52	Obstructive sleep apnoea
		53	Occupational lung disease (various)
		54	Osteoporosis
		55	Other slowly degenerative neurological conditions
		56	Peripheral vascular disease
		57	Personality disorders
		58	Phobias
		59	Physical disabilities
		60	Polycystic ovary disease
		61	Post-traumatic stress
		62	Progressive supranuclear palsy
		63	Psoriasis
		64	Rare disease, genetic disorders
		65	Sarcoidosis
		66	Sensory problems/disabilities (deafness/blindness)
		67	Severe skin conditions
		68	Sickle cell disease
		69	Skin conditions
		70	Sleep disorders
		71	Speech deficits
		72	Spina bifida
		73	Spinal injuries
		74	Stroke/transient ischaemic attacks
		75	Tuberculosis
		76	Urinary Incontinence
		77	Urticarial
